# Supplementary material for: Direct protein-lipid interactions shape the conformational landscape of secondary transporters
Source: Nat Commun. 2018 Oct 8;9:4151. doi: 10.1038/s41467-018-06704-1 (PMC6175955; doi:10.1038/s41467-018-06704-1)
Supplement: Supplementary file 1 — Supplementary Information [file 41467_2018_6704_MOESM1_ESM.pdf]

Direct protein-lipid interactions shape the conformational landscape of secondary transporters

Martens et al.

Supplementary Information

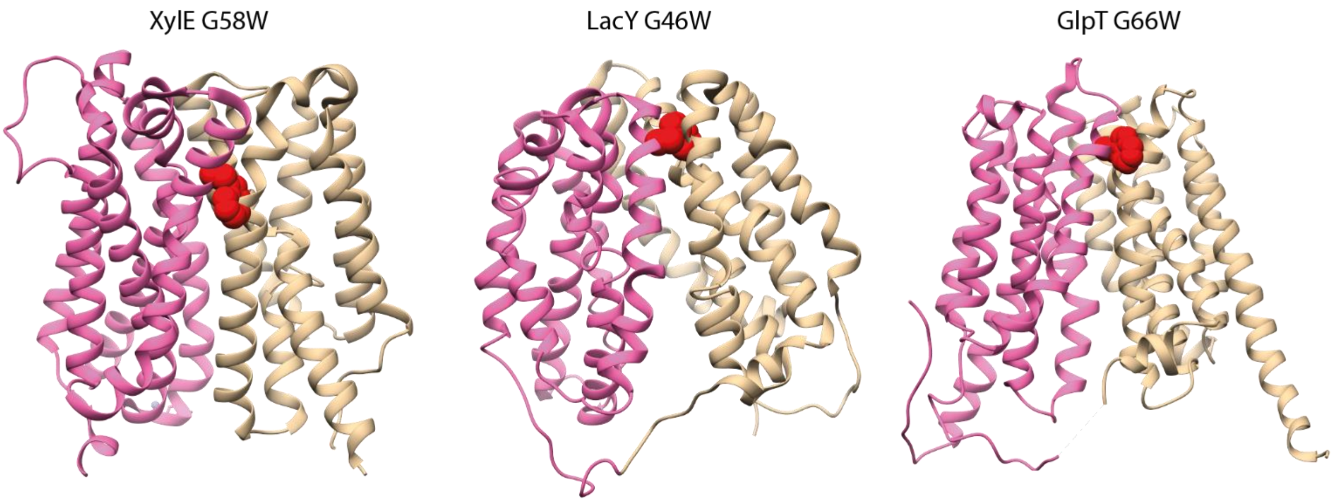

Supplementary Figure 1 . Conformationally restrained mutants of LacY, XylE and GlpT. The location of the tryptophan replacing the native glycine is shown on the inward-facing structures of XylE (PDB: 4QIQ), LacY (PDB: 2V8N) and GlpT (PDB: 1PW4). The N-terminal lobe is colored pink, the C-terminal lobe is colored tan. The tryptophan (red) is located between helix 2 and 11.

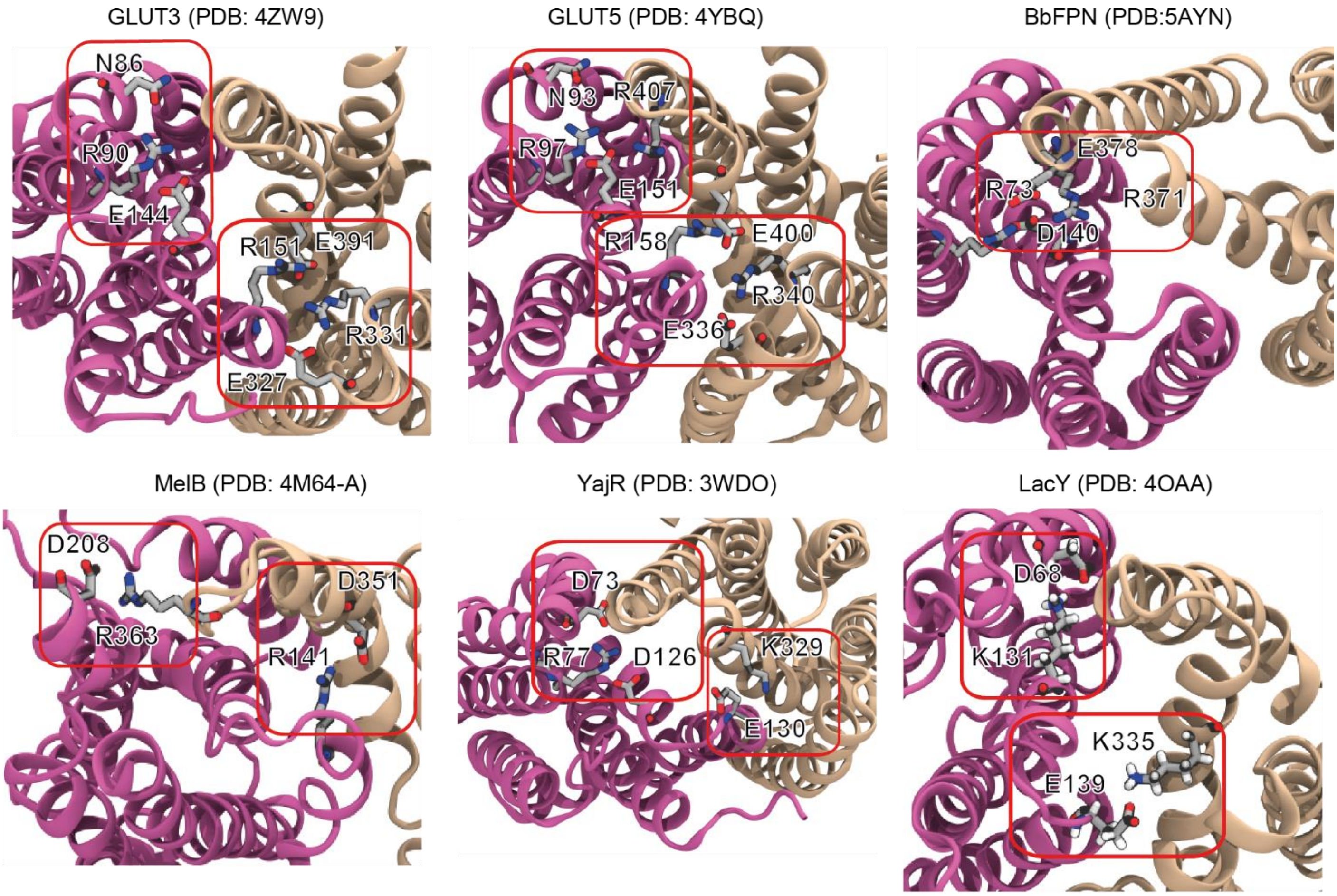

Supplementary Figure 2. Similar charge-relay networks are observed on the crystal structures of various MFS transporters. Snapshots of the cytoplasmic face of 6 MFS transporters crystalized in outward-facing or outward-occluded conformations show the presence of charge networks connecting the N- and C- lobes (pink and tan respectively).

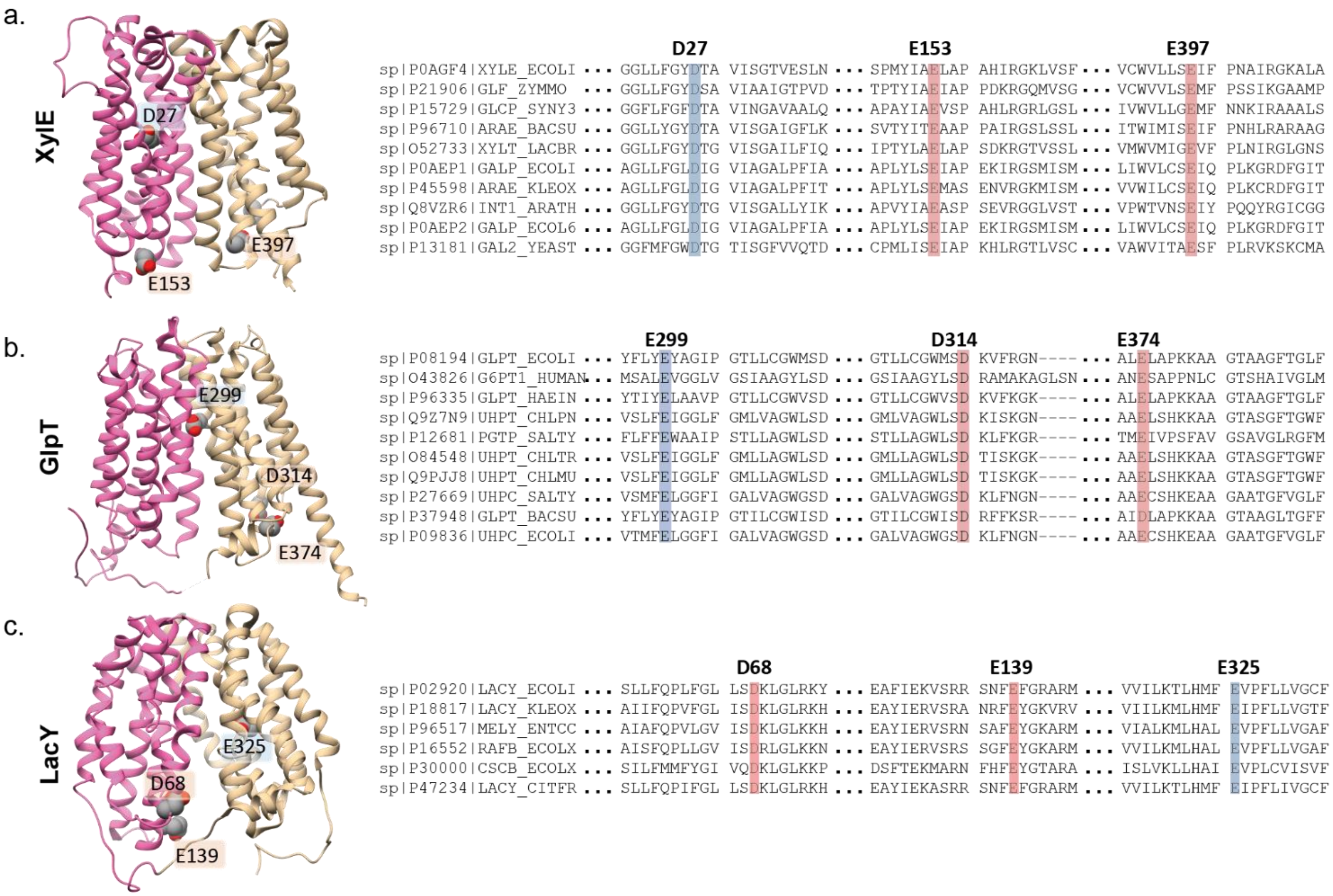

Supplementary Figure 3 . Location of the mutated acidic residues. The conserved acidic residues are shown on the crystal structures of (a) XylE (PDB: 4QIQ), (b) GlpT (PDB: 1PW4) and (c) LacY (PDB:2V8N). The N-terminal lobe is colored pink, the C-terminal lobe is colored tan. Snapshots of the alignment files show the conservation of the residues. Residues part of the charge-relay networks located on the cytoplasmic side are highlighted in red and residues part of salt-bridging interactions in the transmembrane region are highlighted in blue..

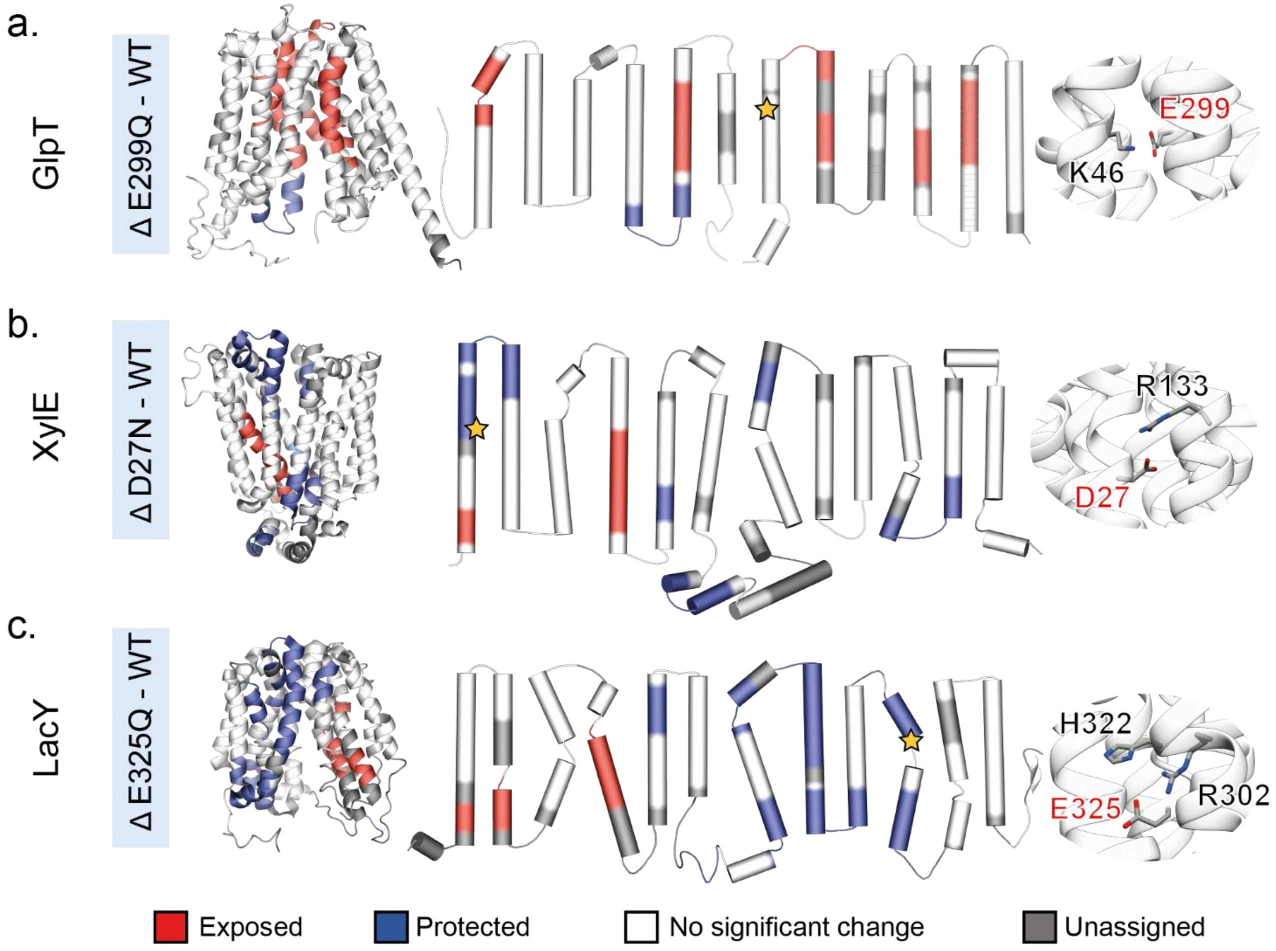

Supplementary Figure 4. Mutation of transmembrane key acidic residue gives rise to various ΔHDX patterns. Carboxylates to amine mutations were used to monitor shifts in the conformational equilibrium. Differential deuterium uptake pattern (ΔHDX) is mapped onto the 3D and topological structure of (a) GlpT, (b) XylE and (c) LacY. The location of the mutation is indicated by a star. An inset on the right shows the salt-bridging interactions. Red and blue colored regions indicate segments containing peptides that become comparatively more deuterated (red) or less deuterated (blue), respectively; white regions indicate that no significant ΔHDX is observed, and grey indicates regions where peptides were not obtained for both the mutant and the WT conditions.

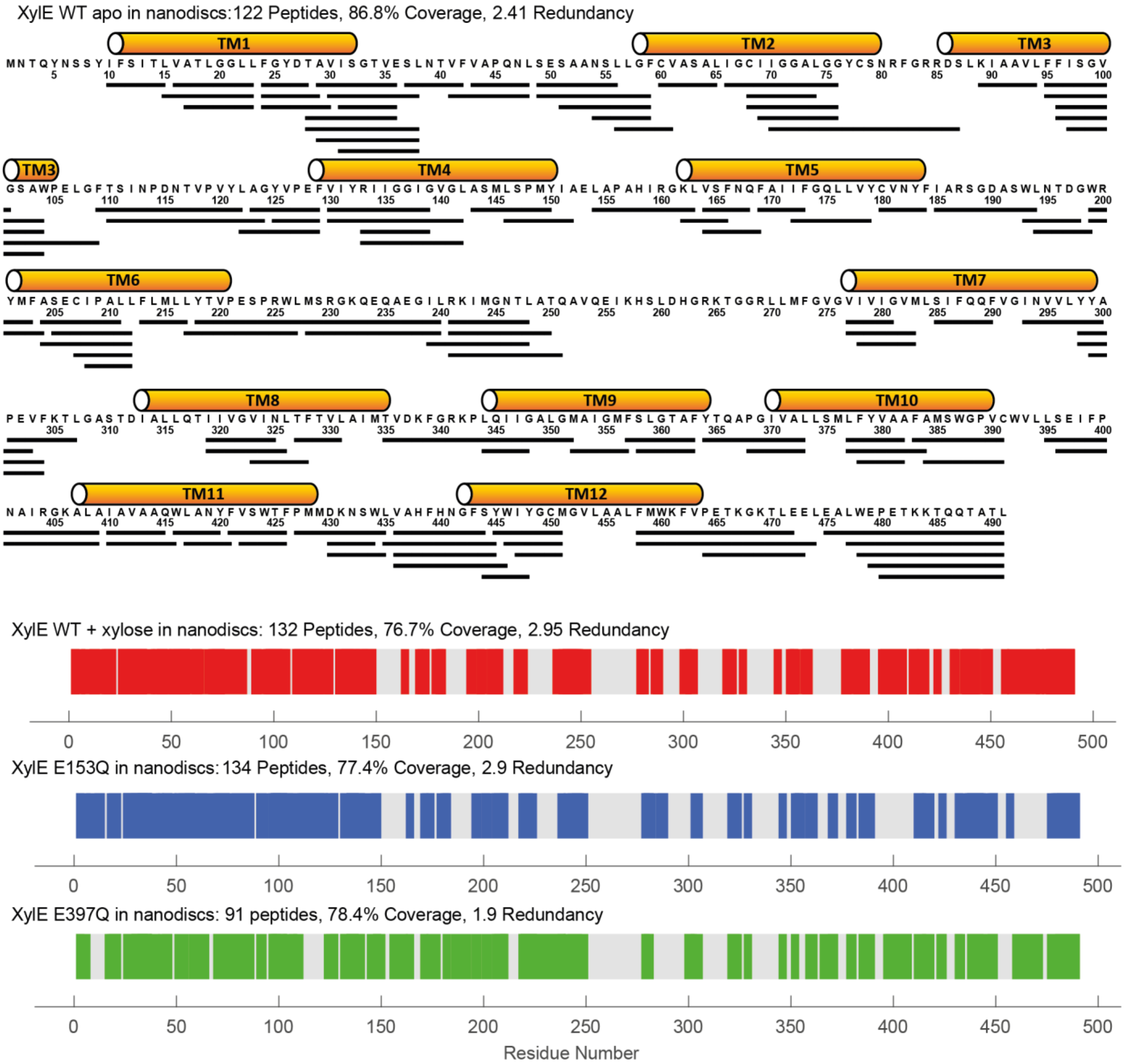

Supplementary Figure 5. Sequence coverage obtained for XylE constructs reconstituted in nanodiscs. Peptides are presented as black bars aligned to their position along the sequence of XylE on a detailed peptide map (top), or as a simplified linear peptide map generated using an in-house software. All peptides were those finalised and output from DynamX HDX analysis software (Waters Corp., Manchester).

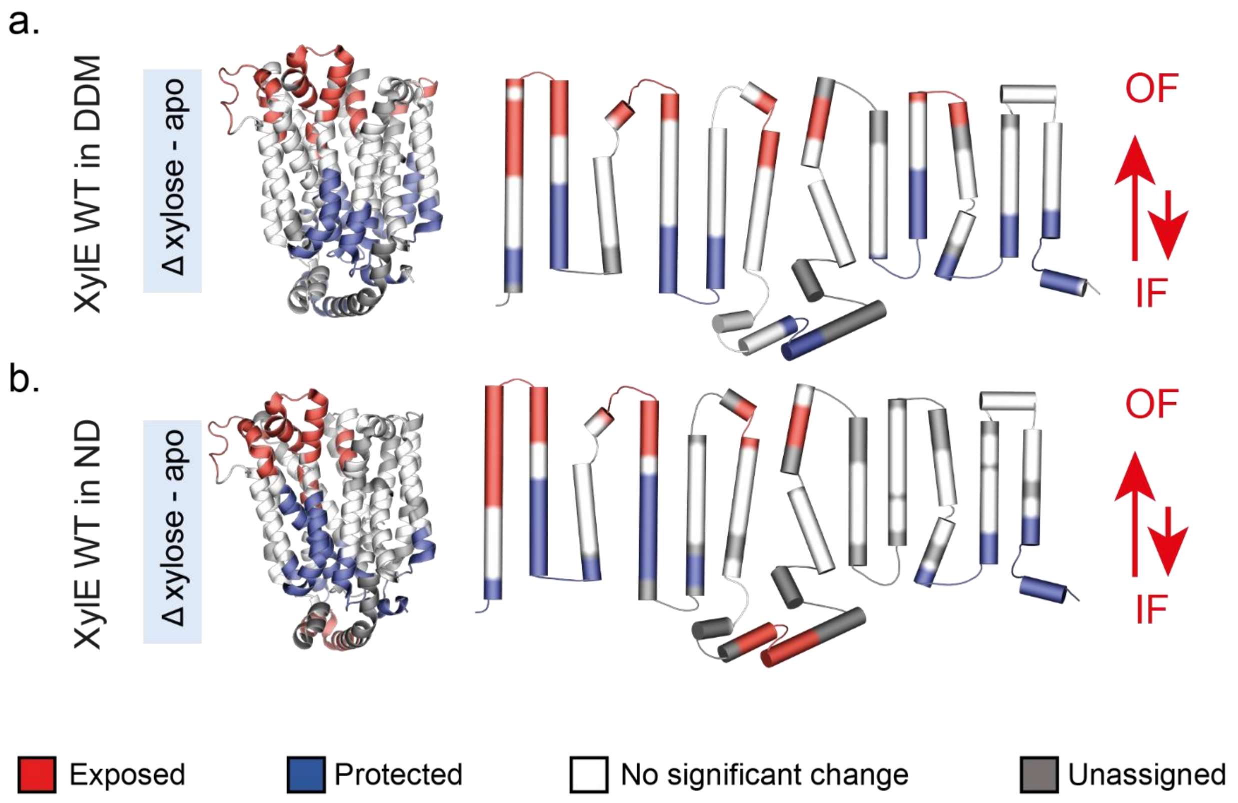

Supplementary Figure 6. Xylose binding shifts the conformational equilibrium toward the OF conformation. ∆HDX between apo XylE vs XylE in the presence of 15mM of xylose mapped onto the 3D and topological structure of XylE. The conformational shift is observed in (a) detergent micelles and (b) in nanodiscs. All measurements were performed in triplicates. The datasets are presented as Woods plots in supplementary Fig.12.

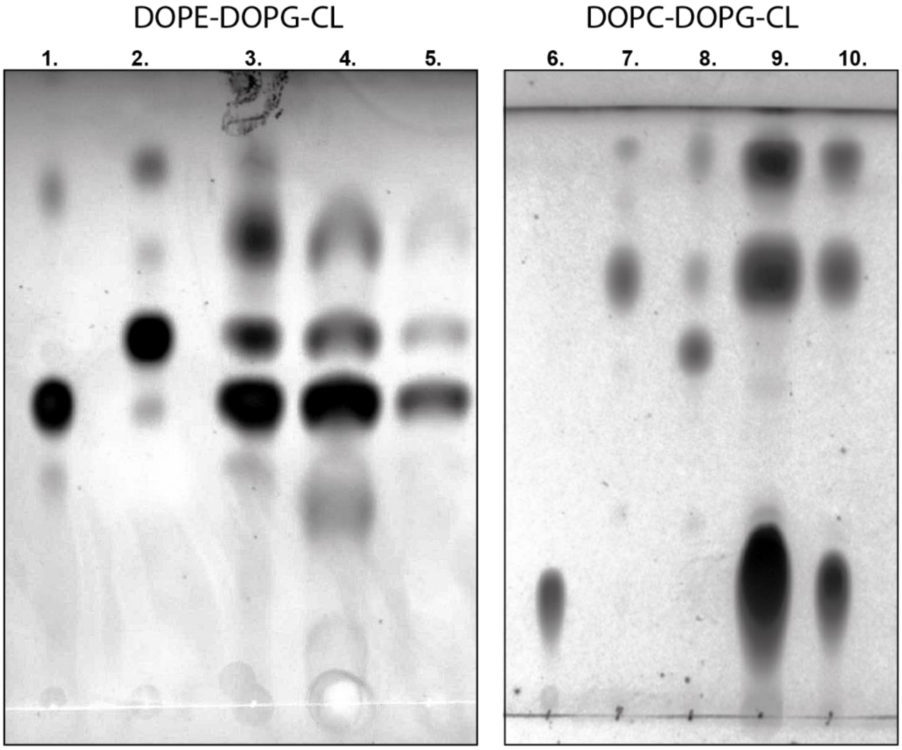

1. reference lipid DOPE

2. reference lipid DOPG

3. mix of reference lipids with DOPE, DOPG, cardiolipin 18:1 (CL)

4. DOPE:DOPG:CL lipid mix used for nanodisc reconstitution

5. lipids extracted from the nanodiscs

6. reference lipid DOPC

7. reference lipid DOPG

8. mix of reference lipids with DOPE, DOPG, cardiolipin 18:1 (CL)

9. DOPC:DOPG:CL lipid mix used for nanodisc reconstitution

10. lipids extracted from the nanodiscs

Supplementary Figure 7. Thin Layer Chromatography of phospholipids starting mixes and nanodiscs. Comparison between lanes 4 and 5 on the left plate (DOPE:PG:CL) and lanes 9 and 10 on the right plate (DOPC:DOPG:CL) shows that the same lipid species are present in both samples.

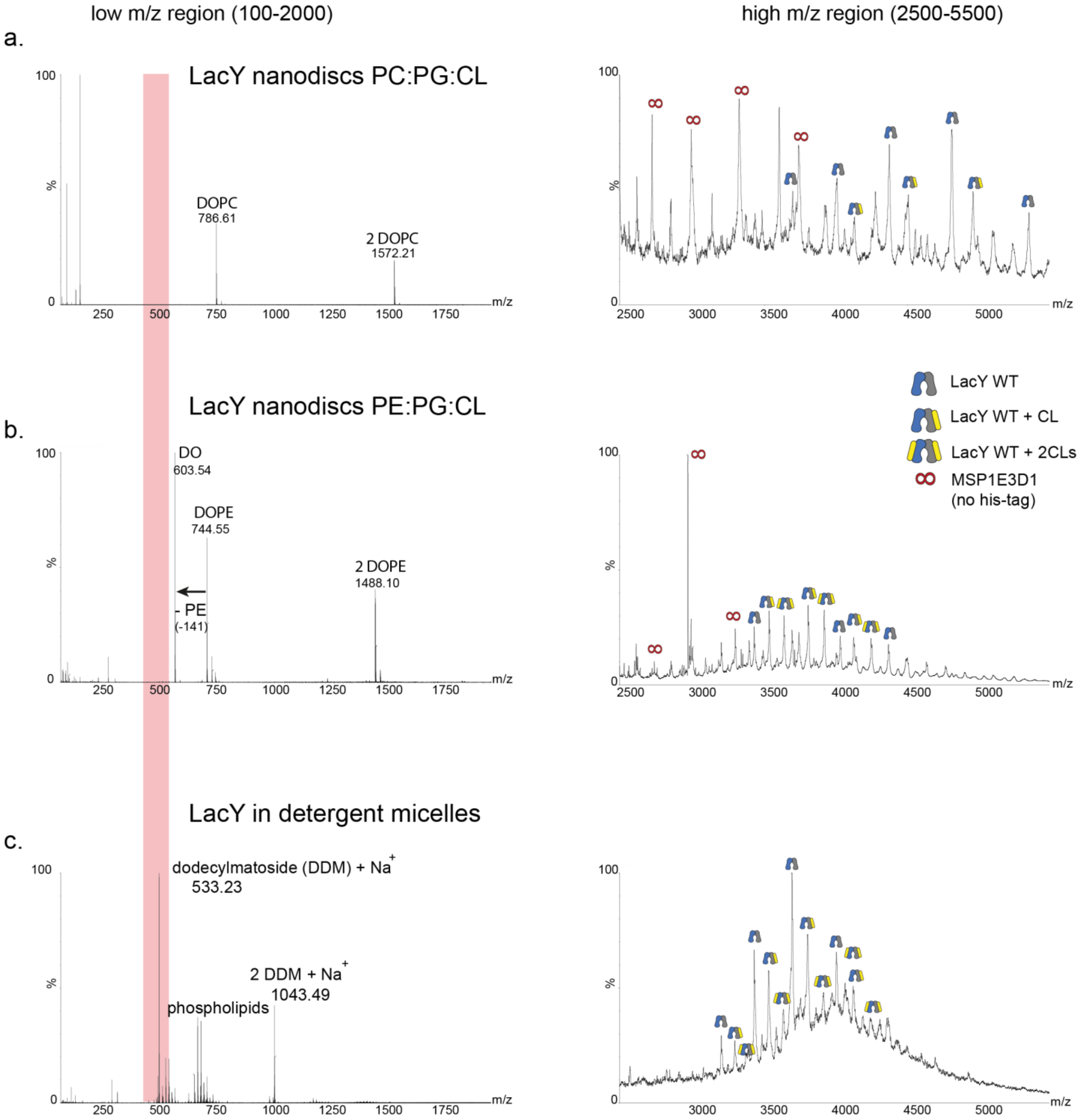

Supplementary Figure 8. Native mass spectrometry shows that no detergent is detectable in nanodisc samples. Mass spectra of (a) LacY in PC nanodiscs, (b) LacY in PE nanodiscs, (c) LacY in detergent micelles. The detergent and free lipids are shown in the low m/z region on the left and the protein with bound lipids is shown in the high m/z region on the right. The position of the DDM peak is indicated by a red rectangle.

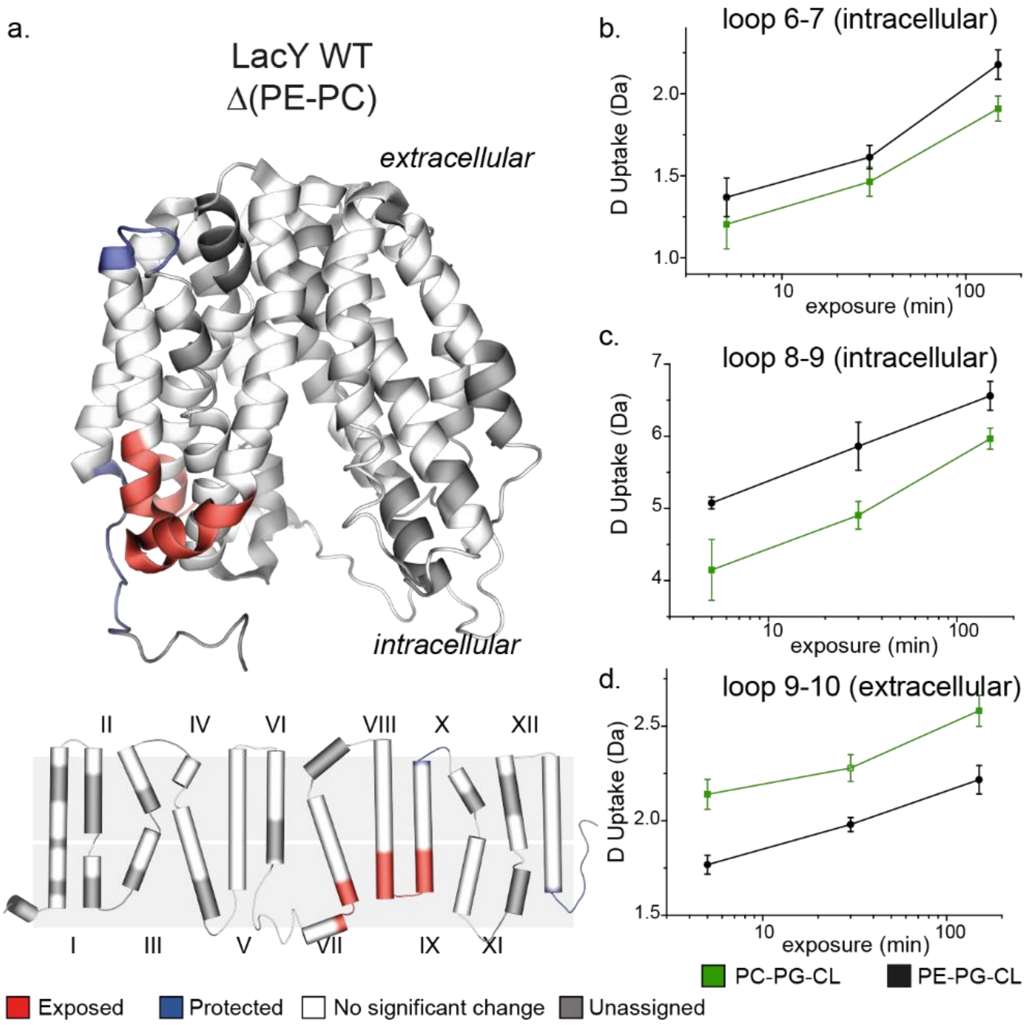

Supplementary Figure 9. HDX-MS of LacY WT in nanodiscs suggests that PE promotes the IF conformation. ΔHDX of WT LacY in DOPE-PG-CL nanodiscs (native-like) minus DOPC-PG-CL (control) nanodiscs mapped on the PDB structure and the topological representation. (b) Representative deuterium uptake plots for peptide 217-224, (c) 279-293, (d) 308-313 in DOPC-PG-CL nanodiscs (green) and DOPE-PG-CL nanodiscs (black). Standard deviations for each time point are plotted as error bars (n=3). All measurements were performed in triplicates.

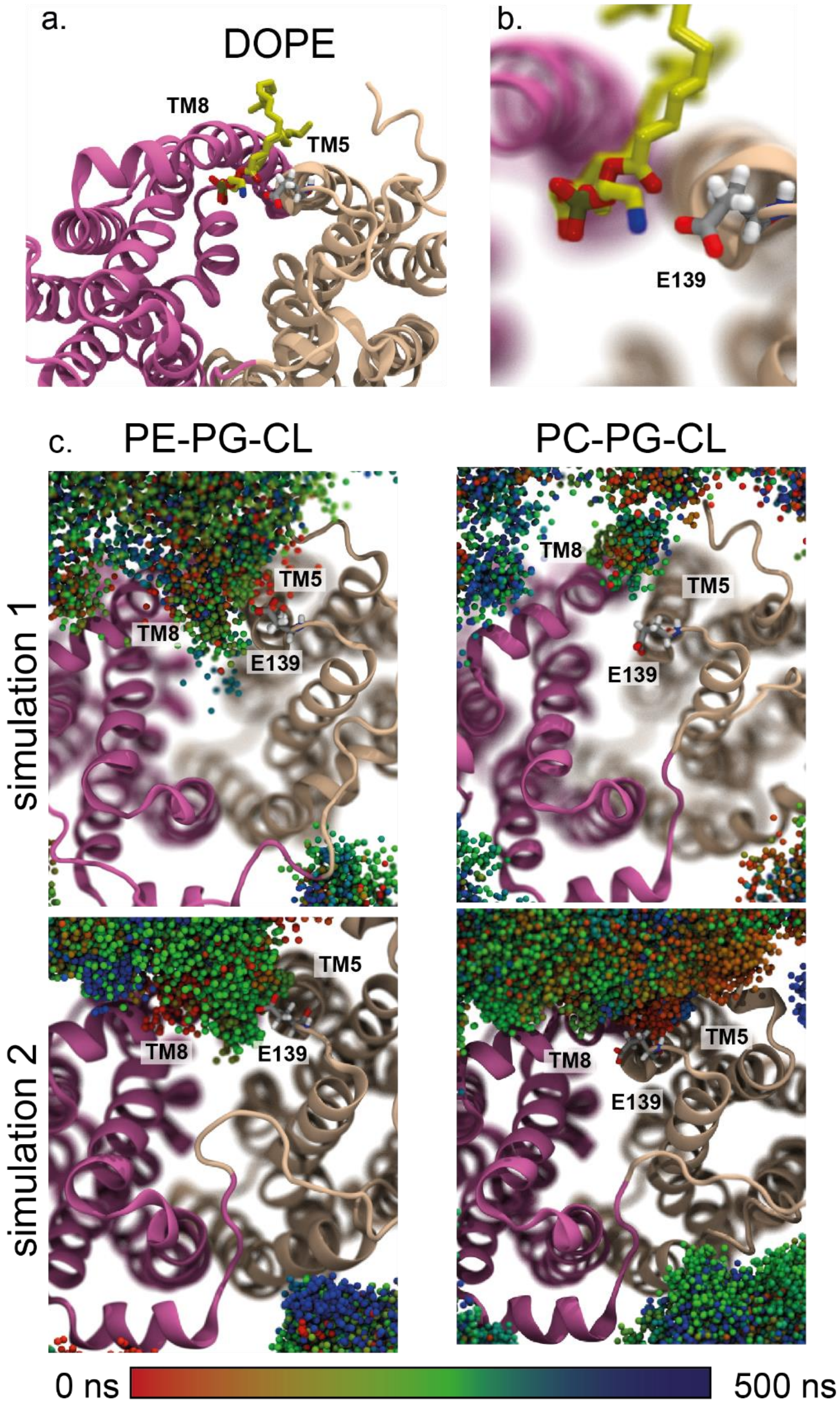

Supplementary Figure 10. MD simulations predict specific interactions between the PE headgroup and acidic residue E139 of LacY (a) Representative snapshot of the entrance of the PE phospholipid into the cytoplasmic space of LacY. Steric hindrance prevents contacts of TM5 and TM8. (b) Close-up of the interaction between the amine headgroup and the acidic residue E139. (c) Location of the phosphorus atoms of the PE/PC lipid molecule in 500 ns trajectories in PE:PG:CL bilayers (left) and PC:PG:CL bilayers (right). The spheres are colour coded: red at t=0 ns and blue at t = 500 ns. PE lipids wedge between the helices but no lipid-protein interaction is observed in PC bilayers.

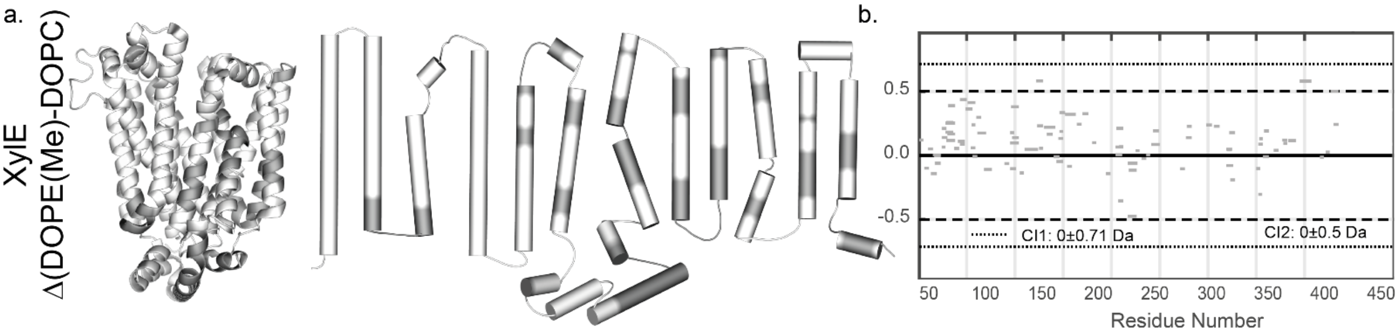

Supplementary Figure 11. HDX-MS of XylE WT in nanodiscs of lipids with different intrinsic curvature shows that curvature properties of the bilayer play a minor role in modulating the conformational equilibrium. ΔHDX of WT LacY in DOPE(Me)$_1$-PG-CL nanodiscs (high curvature) minus DOPC-PG-CL (low curvature) nanodiscs mapped on the PDB structure (4GBY) and the topological representation. (b) Woods plot of differences in relative deuterium uptake (ΔHDX) of XylE. The length of the lines represents the length of the peptide. CI1 and CI2 represent the 99% and 98% confidence interval respectively.

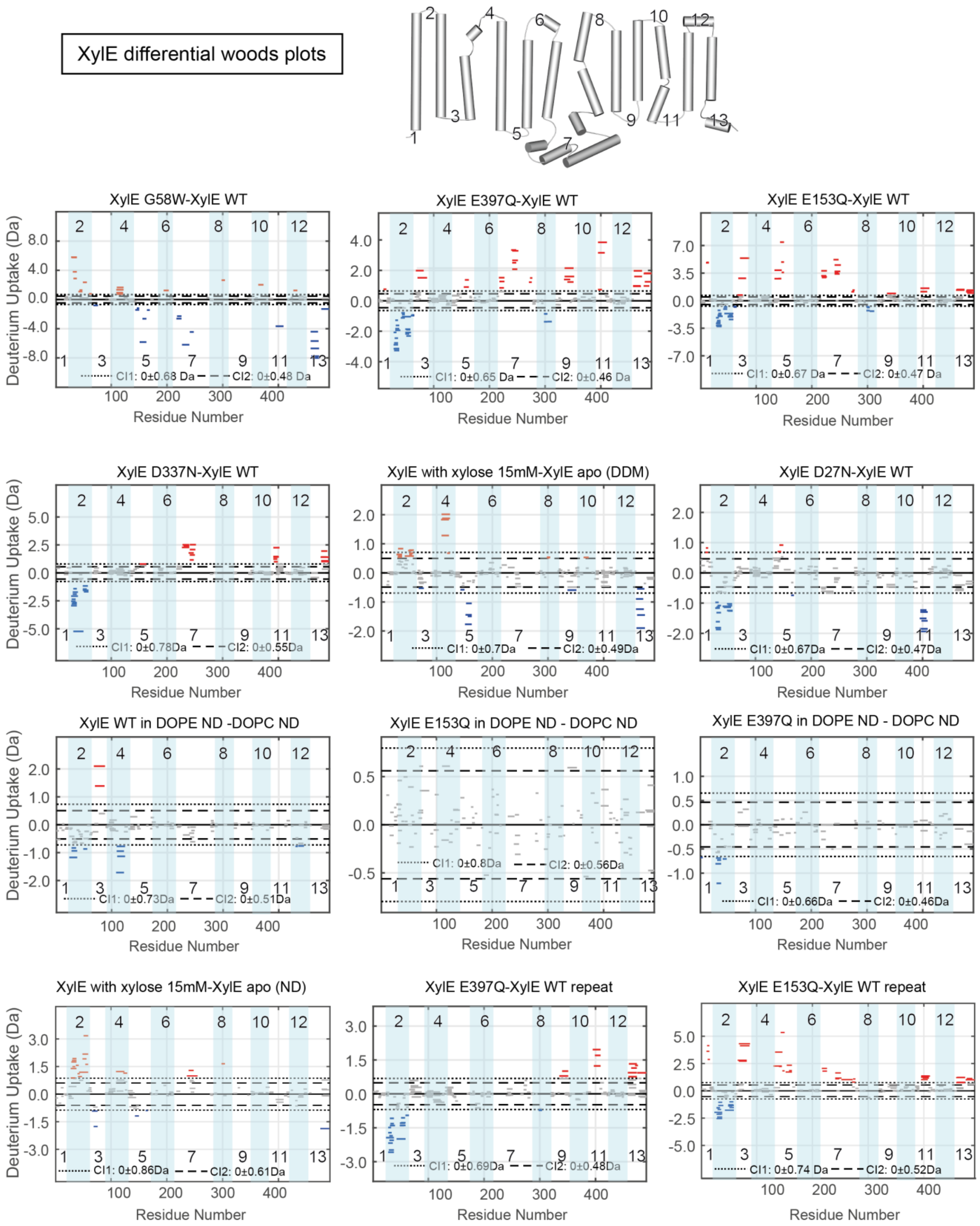

Supplementary Figure 12. Woods plots of differences in relative deuterium uptake (ΔHDX) of XylE. The length of the lines represents the length of the peptide. CI1 and CI2 represent the 99% and 98% confidence interval respectively. The location of the peptides are reported on the topological map of XylE.

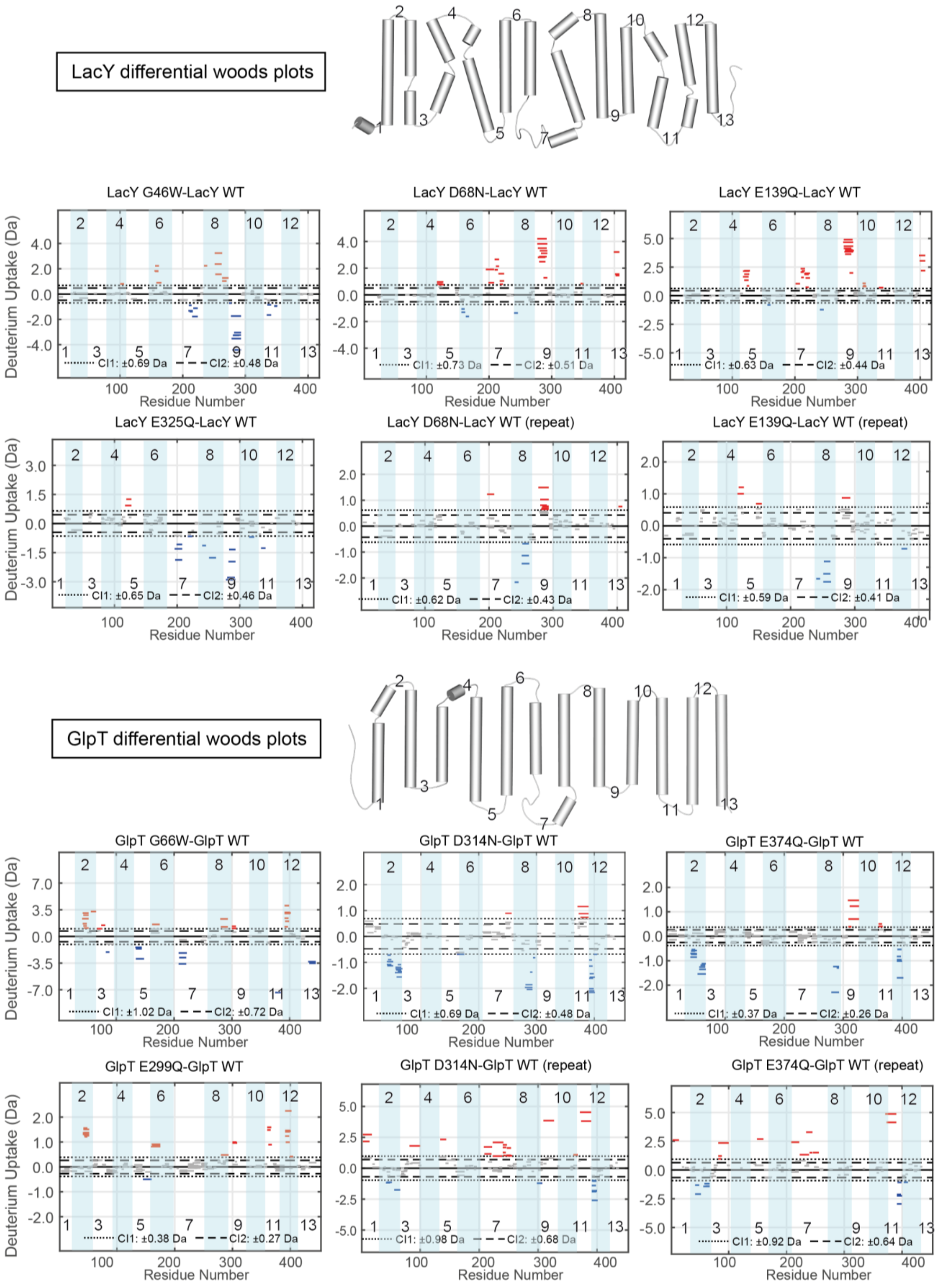

Supplementary Figure 13. Woods plots of differences in relative deuterium uptake (ΔHDX) of LacY and GlpT. The length of the lines represents the length of the peptide. CI1 (dotted lines) and CI2 (dashed lines) represent the 99% and 98% confidence interval respectively. The approximate location of the peptides is reported on the topological map of LacY and GlpT.

Primer name	Primer sequence (5' to 3')
XylE G58W forward	cacgcaaaaccataacagggagttggcagcgg
XylE G58W reverse	ccgctgccaactccctgttatggttttgcgtg
XylE E153Q forward	tgagctggagccagttgcgcaatatacattggc
XylE E153Q reverse	gccaatgtatattgcgcaactggctccagctca
XylE D337N forward	ggcttacgaccaaatttattcaccgtcataattgccaga
XylE D337N reverse	tctggcaattatgacggtgaataaatttggtcgtaagcc
XylE E397Q forward	gcattcgggaagatttgcgacagcagtacccag
XylE E397Q reverse	ctgggtactgctgtcgcaaatcttcccgaatgc
XylE D27N forward	cggaaataacggcggtgttgtagccaaataataaacc
XylE D27N reverse	ggtttattatttggctacaacaccgccgttatttccg
LacY G46W forward	aaccatatcagcaaaagtgatacgtggattatttttgccgctatttctctg
LacY G46W reverse	cagagaaatagcggcaaaaataatccacgtatcacttttgctgatatggtt
LacY D68N forward	atttgcgcagcccgagtttattagaaagcagaccaaacagc
LacY D68N reverse	gctgtttggtctgctttctaataaactcgggctgcgcaaat
LacY E139Q forward	gcgcgaccaaattggaaattactgcgacggc
LacY E139Q reverse	gccgtcgcagtaatttccaatttggtcgcgc
LacY E325Q forward	gcaggaacggtacttgaaacatatgcagcgttttca
LacY E325Q reverse	tgaaaacgctgcatatgtttcaagtaccgttcctgc
GlpT G66W forward	ccgaaagggcaaaccataaatcaccgcgtgagaatccct
GlpT G66W reverse	agggattctcacgcggtgatttatggtttgccctttcgg
GlpT D314N forward	gtgcggctggatgtcgaataaagtcttccgtgg
GlpT D314N reverse	ccacggaagactttattcgacatccagccgcac
GlpT E374Q forward	cttttttcggtgccagctgcagcgcatgcagaccg
GlpT E374Q reverse	cggtctgcatgcgctgcagctggcaccgaaaaaag
GlpT E299Q forward	ggaatacctgcatactgataaaggaagtaggcccaggagg
GlpT E299Q reverse	cctcctgggcctacttcctttatcagtatgcaggtattcc

Supplementary Table 1. DNA primers for the single-point mutants of XylE, LacY and GlpT

Supplementary Methods

Native mass spectrometry

Capillaries for nESI were prepared using a Model P-97 (Sutter Instruments) capillary puller. Capillaries were gold coated using a Q150R S sputter coater (Quorum). Using Micro Bio-Spin 6 columns (Bio-Rad) LacY samples were buffer-exchanged into MS-compatible buffer (300 mM Ammonium acetate (pH 7.0), 0.02% DDM (w/v)) to a final protein concentration of ~15 µM. The individual proteins samples were loaded into gold-coated nanoflow capillaries and introduced into a Synapt G2-Si (Waters) mass spectrometer by nESI. The conditions for maximum peak resolution for LacY samples were: capillary voltage +1.35 kV, sampling cone voltage 15 V, trap collision energy (CE) 125-200 V, transfer CE 75 V, backing pressure 3.88 mbar, trap and transfer pressure (argon) 1.72e^{-2} mbar, ion mobility cell pressure (nitrogen) 2.58 mbar. Mass measurements were calibrated using caesium iodide (100 mg/mL). Spectra were recorded and smoothed using Masslynx 4.1 (Waters) software.

Thin-Layer chromatography

In order to compare the lipid composition of lipid films versus the nanodiscs samples, the lipids were extracted after nanodisc formation according to the method of Bligh and Dyer with some modifications. Briefly, the following procedure was repeated thrice: (i) addition of chloroform, methanol and water to the nanodisc sample and (ii) extraction of the lipid containing chloroform phase upon phase separation. The lipid extracts were then separated on TLC plates. Prior to separation, the plates were washed with a 1:1 chloroform:methanol solution, then immersed in solution A (2.3% boric acid in methanol) and heated at 100C for 15 min. Approximately 100 nmol lipids were loaded to a TLC plate and were separated upon migration in the mobile phase chloroform/methanol/acetic acid at a 65/25/4 ratio (v/v/v). The separations were visualized in the fluorescent blue mode after charring the plate by immersion in solution B (8% phosphoric acid and 10% copper sulfate in water) over the plate and heating at 145 C for 10 mins. The separated spots were compared to reference DOPE, DOPG, DOPC and cardiolipin (18:1) spots.
