# Supplementary material for: Direct protein-lipid interactions shape the conformational landscape of secondary transporters
Source: Nat Commun. 2018 Oct 8;9:4151. doi: 10.1038/s41467-018-06704-1 (PMC6175955; doi:10.1038/s41467-018-06704-1)
Supplement: Supplementary file 3 — Description of Additional Supplementary Files [file 41467_2018_6704_MOESM3_ESM.pdf]

Description of Additional Supplementary Files

File Name: Supplementary Movie 1
Description: Movie rendered using VMD, depicting the interaction of DOPE lipid with the polar residues on the intracellular side of XylE. N-terminal & C-terminal domain are depicted in cartoon representation, colored in pink and tan respectively. The protein residues, ASN 80, ARG 84, GLU 153 (from N-terminal domain) and ARG 404 (from the C-terminal domain) that interact with the lipid molecule are depicted in licorice representation colored by atom type. Movie starts at t=0 ns and ends at t=500 ns and plays at a rate of 25 ns/sec.

File Name: Supplementary Movie 2
Description: Movie rendered using VMD, depicting the interaction of DOPE lipid with charged residues on the intracellular side of LacY. N-terminal & C-terminal domain are depicted in cartoon representation, colored in pink and tan respectively. The protein residue GLU 139 (from the N-terminal domain) that interacts with the lipid molecule is depicted in licorice representation colored by atom type. Movie starts at t=0 ns and ends at t=500 ns and plays at a rate of 25 ns/sec.
